# Identification of a novel *CRYBB2* missense mutation causing congenital autosomal dominant cataract

**Published:** 2012-01-21

**Authors:** Nicole Weisschuh, Sabine Aisenbrey, Bernd Wissinger, Angelika Riess

**Affiliations:** 1 Centre for Ophthalmology, Institute for Ophthalmic Research, Molecular Genetics Laboratory, Tuebingen, Germany; 2 Centre for Ophthalmology, University Eye Hospital, Tuebingen, Germany; 3 Medical Genetics Department, Institute of Human Genetics, Tuebingen, Germany

## Abstract

**Purpose:**

To identify the genetic defect in a four-generation Croatian family presenting with autosomal dominant cataract.

**Methods:**

Genome-wide linkage analysis with 250K single nucleotide polymorphism (SNP) arrays was performed using DNA from one unaffected and seven affected individuals. Mutation screening of candidate genes was performed by bidirectional Sanger sequencing.

**Results:**

Evidence for linkage was observed for eight genomic regions. Among these was a locus on chromosome 22 which encompasses the β-crystallin gene cluster. This cluster includes four genes, namely beta-crystallin B1 (*CRYBB1*), beta-crystallin B2 (*CRYBB2*), beta-crystallin B3 (*CRYBB3*), and beta-crystallin A4 (*CRYBA4*). A novel sequence variant was found in the *CRYBB2* gene (p.Arg188His). This variant cosegregated with the disease phenotype in all affected individuals but was not present in the unaffected family members and 100 healthy control subjects.

**Conclusions:**

We report a novel missense mutation, p.Arg188His, in CRYBB2 associated with congenital cataract in a family of Croatian origin. This variant is the most COOH-terminal missense mutation in *CRYBB2* that has been identified so far.

## Introduction

**Figure 1:**
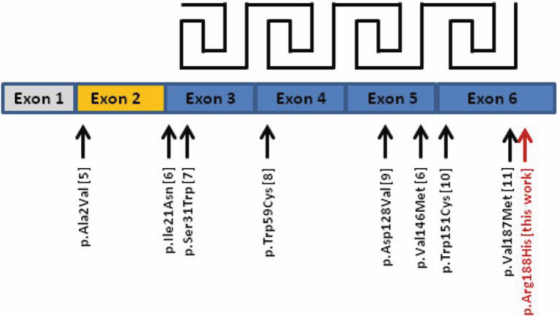
Schematic overview of gene structure and missense mutations found in *CRYBB2*. The *CRYBB2* gene consists of six exons; each of the exons 3 to 6 encodes one Greek Key motif. The mutation identfied in this study is the most COOH-terminal identified so far and highlighted in red.

Congenital cataracts occur with a frequency of 30:100,000 in developed countries and most of them are caused by mutations in genes that are associated with the lens or surrounding ocular tissues [1]. Congenital cataracts often follow Mendelian inheritance patterns, with autosomal dominant traits being more common than autosomal recessive and X-linked traits [2]. The transparency of the lens results from a tight and highly organized packing of lens proteins which enhance refraction without scattering light. Of the lens proteins, the crystallins are particularly abundant. Three major types of crystallins are found in the mammalian lens, namely the α-, β- and γ-crystallins. According to the Human Gene Mutation Database (HGMD), mutations in genes encoding for the different crystallins are found in 50% of the cataract families for whom the mutant gene could be identified, highlighting their relevance in cataract formation. Mutations are found in all three crystallin types. Among the genes in which mutations are most frequently found is beta-crystallin B2 (*CRYBB2*; 13 HGMD entries), together with alpha-crystallin A (*CRYAA*; 12 HGMD entries), alpha-crystallin B (*CRYAB*; 16 HGMD entries), and gamma-crystallin D (*CRYGD*; 16 HGMD entries). βB2-crystallin is the most abundant and most soluble β-crystallin in the lens [3]. βB2-, like all β-crystallins, is characterized by so-called Greek key motifs which refer to the arrangement of antiparallel beta sheets in the protein. Each motif consists of four β-strands and four motifs form four β-sheets. It is hypothesized that the Greek key motif facilitates the dense packaging of β-crystallins in the lens [4]. In the βB2-crystallin, four Greek key motifs are encoded by exons 3–6 (Figure 1). So far, eight missense mutations have been identified in *CRYBB2*; all of them in families with autosomal dominant cataract formation [5-11].

## Methods

### Clinical evaluation and DNA specimens

A four-generation family presenting with autosomal dominant congenital cataracts was ascertained from the Medical Genetics Department of the Institute of Human Genetics, Tuebingen, Germany. The study adhered to the tenets of the Declaration of Helsinki. After informed consent, consistent with the Institutional Review Board approval, twelve individuals participated in the study, seven affected and five unaffected.

Ophthalmic examination included best corrected visual acuity, cover test, pupillary reaction, biomicroscopy of the anterior chamber, indirect fundoscopy, intraocular pressure (Eyecare non-contact tonometry), and ophthalmic ultrasonography (B-mode).

Genomic DNA was extracted from peripheral blood leukocytes using standard protocols.

### SNP genotyping and linkage analyses

We performed a genome-wide linkage analysis using Affymetrix GeneChip Human Mapping 250K single nucleotide polymorphism (SNP) arrays (Affymetrix, Inc., Santa Clara, CA) and genomic DNA samples from eight individuals from one family. Linkage analysis was performed assuming autosomal dominant inheritance, full penetrance and a disease gene frequency of 0.001. Multipoint logarithm of odds (LOD) scores were calculated using ALLEGRO [12] implemented in easyLINKAGE software [13].

### Sanger sequencing

To screen the coding regions of *CRYBB1*, *CRYBB2*, *CRYBB3*, and *CRYBA4*, gene specific PCR primers were designed flanking each exon and intron-exon junctions. Primer sequences are given in Appendix 1. For each PCR reaction, 20 ng of genomic DNA in a total volume of 25 µl using 10 pmol of forward and reverse primer was used together with buffer (100 mM Tris pH 8.9, 500 mM KCl, 15 mM MgCl_2_, 0.01% gelatin), 10 mM dNTPs and 1 U SB Taq Polymerase I (ATG Biosynthetics, Merzhausen, Germany). PCR was performed on a thermocycler using the following conditions: 3 min at 95 °C followed by 35 cycles of 95 °C for 15 s, 55-60 °C for 15 s, and 72 °C for 30 s and one extension step at 72 °C for 7 min. PCR fragments were purified by ExoSAP-IT treatment (USB, Cleveland, OH), sequenced using Big Dye Termination chemistry (Applied Biosystems [ABI], Weiterstadt, Germany) and products separated on a DNA capillary sequencer (ABI 3100 genetic analyzer; ABI).

### Restriction fragment length polymorphism assay

The novel missense mutation in the *CRYBB2* gene identified in this study was evaluated by analysis of 100 healthy control subjects (200 chromosomes) applying a PCR/restriction fragment length polymorphism (RFLP) assay. The G→A transition at codon 188 (Arg188His) of *CRYBB2* results in the gain of an ApaLI restriction site. The respective fragment harboring the missense mutation was amplified from family members and from control subjects. An aliquot of each amplicon was digested with ApaLI (New England Biotechnology [NEB], Beverly, MA). All restriction digests were analyzed on a 4% agarose gel.

### In-silico protein analysis

Biophysical predictions of the altered protein were analyzed using the Protean 3D software (DNAStar, Madison, WI). For protein structure predictions, we used the SWISS-Pdb viewer [14] for automated homology protein modeling.

## Results

**Figure 2:**
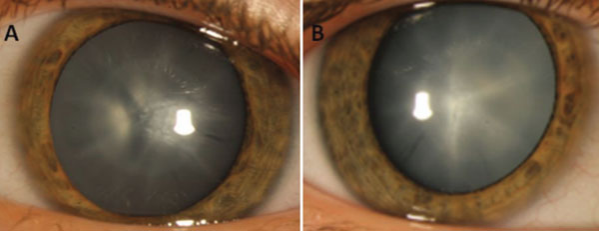
Slit lamp photographs of an affected individual (IV:1) showing anterior axial embryonal nuclear cataract in both eyes (**A**, right eye; **B**, left eye).

**Figure 3:**
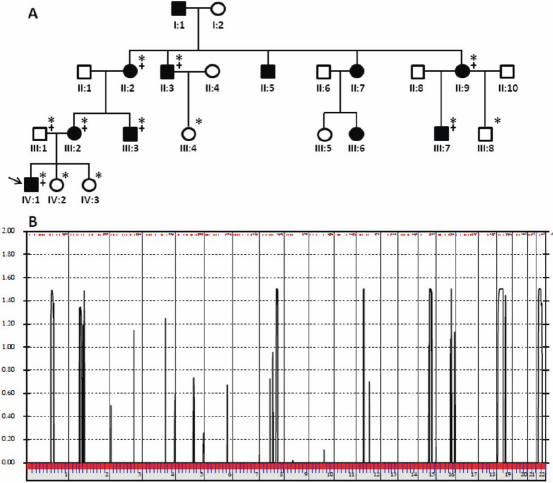
Results of genome-wide linkage analysis. **A**: Family pedigree. The family history revealed eleven affected members in four generations. Squares and circles symbolize males and females, respectively. Clear and blackened symbols denote unaffected and affected individuals. Family members participating in this study are indicated by an asterisk. Those included in the genome-wide scan are also marked with “+.” The arrow points to the index patient. **B**: Graphical view of multipoint genome-wide LOD scores calculated with ALLEGRO using SNP chip data from family members marked with “+” on Figure 3A. The linkage interval on chromosome 22 encompasses the β-crystallin gene cluster.

We have identified a four-generation family of Croatian origin with a diagnosis of congenital cataract in seven family members. Opacification of the lens was bilateral in all affected subjects except for subject III:7 who presented with only one affected eye. Based on the presence of affected individuals in each of the four generations and male to male transmission, autosomal dominant inheritance was evident. Photo documentation of the lens could only be ascertained from the youngest patient IV:1 since all other affected individuals in this family had already had cataract extraction. The photographs (Figure 2) show anterior axial embryonal nuclear cataract without additional pathological findings of the anterior or posterior chamber structures. Both eyes are similarly affected. A pedigree of the family is given in Figure 3A.

**Figure 4:**
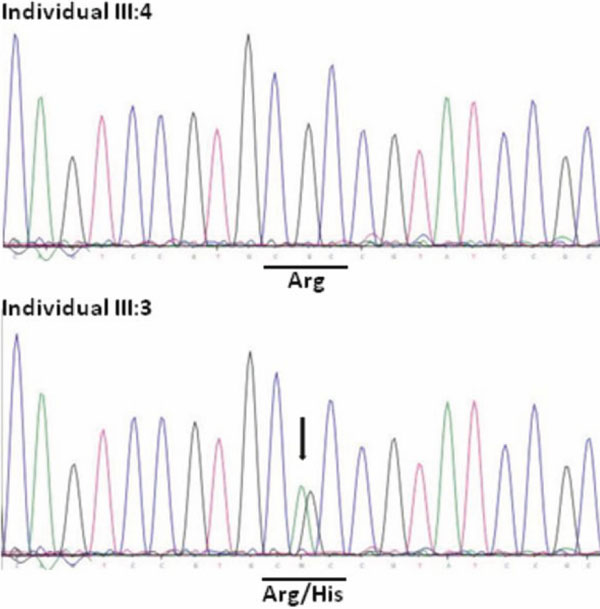
Forward sequence analysis of exon 6 of the *CRYBB2* gene. The arrow indicates the c.563G→A transition. Individual III:4 is normal (upper panel) whereas individual III:3 is affected (lower panel). The encoded amino acid at codon 188 (underlined) is indicated, CGC encodes arginine (Arg), CAC encodes histidine (His).

**Figure 5:**
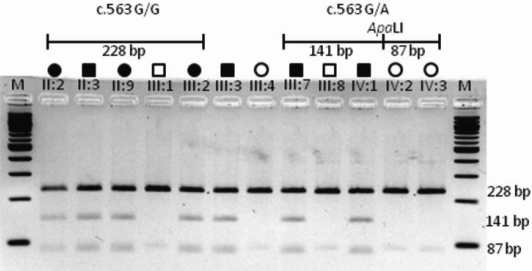
Restriction fragment length analysis showing gain of an ApaLI site that cosegregates only with affected family members heterozygous for the c.563G→A transition. Control subjects were analyzed accordingly (data not shown). M, size marker (100 bp ladder, NEB).

**Figure 6:**
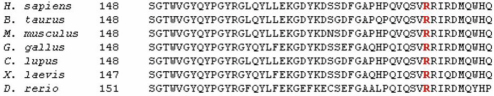
Amino acid alignment of βB2-crystallin shows evolutionary conservation of the residue that is affected by the novel mutation found in this study. Partial amino acid sequences of βB2-crystallin orthologs from seven different species were aligned to show possible conservation. The affected residue is highlighted in red.

**Figure 7:**
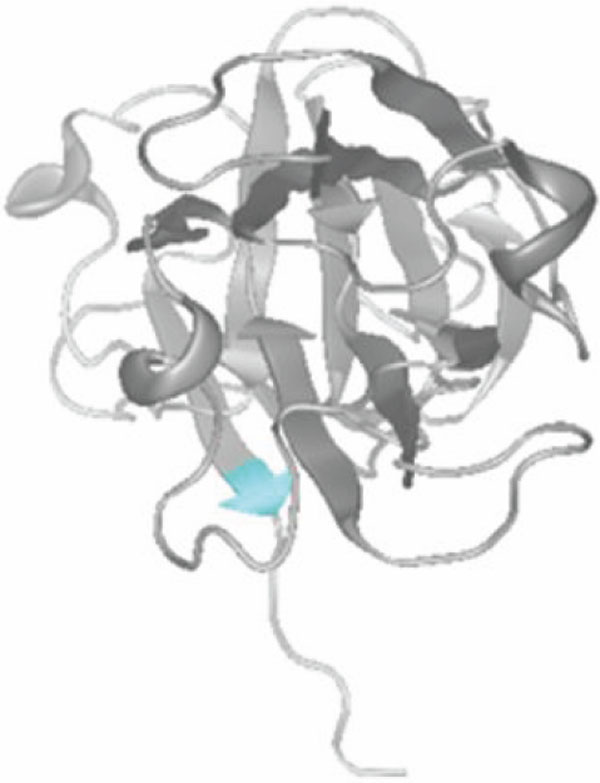
Modeled structure of βB2-crystallin. The mutation described in this study leads to the replacement of arginine 188 (highlighted in blue) by histidine. The Arg188 is located in the β4-sheet of the fourth Greek key motif of the protein.

We performed a genome-wide linkage analysis with 250K SNP arrays using DNA from one unaffected and seven affected individuals. Using multipoint analysis, we observed evidence for linkage to eight genomic regions on chromosomes 1q22-q24, 2p11-p16, 8q22-q24, 12p11-q12, 15q24-q26, 16q23-q24, 19p13, and 22q11-q13, respectively, with LOD score values greater than 1 (Figure 3B). Since the locus defined on chromosome 22 encompasses the β-crystallin gene cluster (*CRYBB1*, *CRYBB2*, *CRYBB3*, and *CRYBA4*), we performed a mutation screening of these four genes using bidirectional Sanger sequencing. No putative pathogenic variants were identified in the coding regions of *CRYBB1*, *CRYBB3* and *CRYBA4*, respectively. Sequencing of exon 6 of *CRYBB2* revealed a heterozygous transition from A→G at codon 188 (Figure 4). This missense mutation segregated with all affected members in the family, but was not detected in 100 unrelated healthy controls and unaffected pedigree members as shown by restriction fragment length analysis (Figure 5). The nucleotide substitution replaces an evolutionarily highly conserved arginine with histidine at amino acid position 188 (p.Arg188His) in the fourth Greek key motif of βB2 crystallin (Figure 6 and Figure 7).

**Figure 8:**
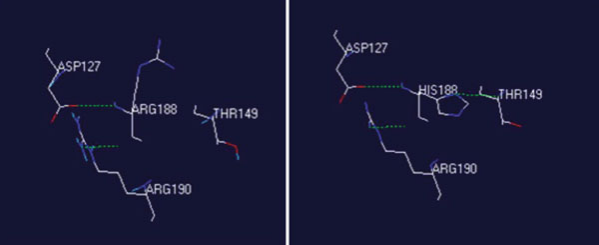
Molecular modeling of the effect of mutation p.Arg188His. Hydrogen-bonding patterns of the normal βB2-crystallin (left) and the mutant βB2-crystallin (right) are shown by dashed lines (green).

As suggested by Polyphen analysis, the mutation p.Arg188His is predicted to be “probably damaging.” However, this prediction is based solely on the conservation of orthologous protein sequences and does not take into account the effect of an amino acid exchange on protein structure. Histidine is less likely to be positively charged than arginine because of its more acidic pK_a_, therefore an effect on protein charge can be assumed. Both the CRYBB2 wildtype protein as well as the p.Arg188His CRYBB2 protein was analyzed using the Protean 3D software (DNAStar) to compare secondary structural characteristics and physicochemical properties. While hydrophilicity is only slightly changed, the mutant protein is characterized by a different charge and a change in surface probability (data not shown). Changes in the secondary structure are predicted as follows: the Arg-His exchange causes the formation of a new hydrogen bond between histidine and threonine at position 149 (Figure 8).

## Discussion

In this study, we have identified a novel causative mutation p.Arg188His in CRYBB2 in a four-generation family of Croatian origin affected with autosomal dominant congenital cataract. The disease gene co-localizes with one of few maximum genome-wide linkage signals in this family.

The correct association and supramolecular assembly of lens crystallins are crucial for lens transparency. βB2-crystallin is the major component of β-crystallin. In the lens, βB2-crystallin forms hetero-oligomers with other β-crystallins [15,16], an interaction that is mediated by β strands [17]. The mutation we identified is located in exon 6 of *CRYBB2*, more precisely in the β4-sheet of the fourth Greek key motif of the protein. The mutation replaces arginine with histidine at position 188 and is the most COOH-terminal missense mutation in *CRYBB2* that has been identified so far. Mothobi and colleagues [11] identified a sequence variant in a Basotho family with congenital nuclear cataract leading to an exchange of the amino acid located directly before position 188 (p.Val187Met), highlighting the functional importance of this region in βB2-crystallin. In the mouse, three mutant alleles of *Crybb2* cause progressive cataracts which all affect exon 6 and therefore the fourth Greek key motif [18-20].

CRYBB2 belongs to the most abundant crystallins in the lens and therefore plays a key role in maintaining lens transparency. Most of the mutations that have been identified in *CRYBB2* are missense mutations that lead to amino acid substitutions (Figure 1). The replacement of Arg188 with a histidine residue as deduced from the mutation in our family is predicted to result in a significant change in the conformation of neighboring residues and in an alteration in the hydrogen bonding pattern as demonstrated in Figure 8. We hypothesize that p.Arg188His, which is located in the β4-sheet, impairs dimerization of the CRYBB2 protein upon the formation of this additional hydrogen bond, thereby leading to lens opacity.

In summary, we identified a novel missense mutation in CRYBB2 (p.Arg188His) that is associated with autosomal dominant congenital cataract in a four-generation Croatian family. The Arg188His is the most COOH-terminal missense mutation in CRYBB2 that has been identified so far and presumably affects the formation of the fourth Greek key motif of the βB2-crystallin. Further biophysical studies are necessary to evaluate the precise molecular mechanism caused by the p.Arg188His mutation.

## Appendix 1.

Appendix 1. Primer sequences for PCR amplification of *CRYBB1*, *CRYBB2*, *CRYBB3*, and *CRYBA4*. To access the data, click or select the words “Appendix 1.” This will initiate the download of a compressed (pdf) archive that contains the file.
